# Iatrogenic Thyrotoxicosis and Corticosteroid-Triggered Hypokalemic Periodic Paralysis in a Patient With Treated Hypothyroidism

**DOI:** 10.7759/cureus.109603

**Published:** 2026-05-25

**Authors:** Gabrielle Brini, Kyle Sharron, Zaineb Ahmad, Chidimma Madu

**Affiliations:** 1 Diagnostic Radiology, Touro College of Osteopathic Medicine, Middletown, USA; 2 Neurology, Touro College of Osteopathic Medicine, Middletown, USA; 3 Internal Medicine, Touro College of Osteopathic Medicine, Middletown, USA; 4 Psychiatry, Garnet Health, Middletown, USA

**Keywords:** hypokalemia, hypokalemic periodic paralysis (hpp), levothyroxine, steroids, thyrotoxicosis

## Abstract

Hypokalemic periodic paralysis (HPP) is a rare disorder characterized by episodic weakness with hypokalemia, most commonly associated with thyrotoxic periodic paralysis (TPP) in the setting of endogenous hyperthyroidism. We present a unique case of a 32-year-old male patient with a history of hypothyroidism who developed recurrent episodes of acute paralysis due to iatrogenic thyrotoxicosis from levothyroxine over-replacement. Notably, each episode occurred shortly after corticosteroid administration, suggesting a dual-trigger mechanism.

The patient presented with profound, predominantly proximal bilateral extremity weakness with greater upper limb involvement on formal examination (2/5 proximally, an uncommon distribution in TPP) alongside severe hypokalemia (1.8-1.9 mmol/L). Laboratory evaluation revealed suppressed thyroid-stimulating hormone (TSH), and symptoms resolved rapidly with potassium repletion and supportive care. Discontinuation of levothyroxine resulted in clinical stabilization without recurrence during hospitalization.

This case highlights an uncommon etiology of TPP due to exogenous thyroid hormone excess and underscores the role of corticosteroids as a potential precipitating factor. Recognition of this interaction is critical for timely diagnosis, prevention of recurrence, and appropriate management. Clinicians should maintain a high index of suspicion for TPP in patients presenting with acute weakness and hypokalemia, even in the absence of known hyperthyroidism.

## Introduction

Hypokalemic periodic paralysis (HPP) is a rare neuromuscular disorder characterized by episodic weakness or paralysis coupled with a shift of potassium into skeletal muscle cells, which results in hypokalemia [1]. Among the different types of acquired HPP, thyrotoxic periodic paralysis (TPP) is the most clinically significant etiology [1]. Despite a higher incidence of thyrotoxicosis in women, TPP occurs predominantly in men, with a male-to-female ratio of approximately 20:1 [2] and the highest prevalence of cases occurring in Asian populations [2].

The underlying pathophysiological mechanism of TPP is excessive Na⁺/K⁺-ATPase pump activity in skeletal muscle [3]. The mechanism of clinical paralysis involves thyroid hormone stimulating Na⁺/K⁺-ATPase gene transcription and increasing beta-adrenergic receptor sensitivity [3]. This leads to an intracellular shift of potassium, causing hyperpolarization of the muscle cell membranes, ultimately resulting in clinical weakness or paralysis depending on the severity [3]. Studies have shown that TPP occurs from a combination of genetic susceptibility, thyrotoxicosis, and environmental factors [4]. 

In susceptible individuals, several factors can precipitate an acute attack, including infection, carbohydrate loading, alcohol, and medications [5]. Corticosteroids are an important but often overlooked trigger [5]. Glucocorticoids promote hypokalemia through mineralocorticoid activity and can cause hyperglycemia and hyperinsulinemia, which can further increase intracellular potassium uptake and can precipitate TPP episodes [6]. We present a case of recurrent TPP in a patient with treated hypothyroidism in whom iatrogenic thyrotoxicosis from levothyroxine overreplacement, combined with repeated corticosteroid injections, potentially produced a dual-trigger mechanism for episodic paralysis. Notably, this case exemplifies a fully reversible cause of acute paralysis, underscoring the importance of identifying and treating the underlying etiology and highlighting a critical but uncommon clinical interaction.

## Case presentation

A 32-year-old male patient with hypothyroidism on levothyroxine (150 mcg daily) presented with acute bilateral upper and lower extremity weakness beginning approximately four hours after receiving an intra-articular triamcinolone acetonide 40 mg injection for a left knee meniscal injury. He reported a near-identical episode six weeks prior, following the same corticosteroid agent and dose, which resolved spontaneously within 24 hours. His levothyroxine dose had not been adjusted between episodes, and he had not undergone interval thyroid function testing.

On admission, physical examination revealed profound proximal-predominant weakness in all four extremities, graded 2/5 proximally and 3-4/5 distally, with diffusely diminished but present deep tendon reflexes. Sensation was intact throughout. There was no cranial nerve or bulbar involvement. Vital signs were stable. The pattern of symmetric proximal weakness without sensory deficit or upper motor neuron signs was consistent with a metabolic or neuromuscular etiology rather than a structural lesion. Laboratory findings were significant for severe hypokalemia (serum potassium 1.9 mmol/L), mild hypophosphatemia (phosphate 2.1 mg/dL), leukocytosis (WBC 17.7 × 10³/µL), and elevated liver enzymes, including alanine aminotransferase and alkaline phosphatase (Table 1). Creatine kinase was within normal limits, excluding rhabdomyolysis. Leukocytosis was attributed to corticosteroid-induced demargination, supported by negative blood cultures and an unremarkable urinalysis. An electrocardiogram demonstrated a prolonged QTc of 498 ms with prominent U-waves; no arrhythmias were identified on continuous telemetry. Thyroid function testing demonstrated a suppressed thyroid-stimulating hormone (TSH; <0.100 mIU/L), an elevated free triiodothyronine (T3; 5.8 pg/mL; reference range 2.3-4.2 pg/mL), and a free thyroxine (T4) of 1.43 ng/dL within the population reference range, though notably elevated compared to the patient's own prior value of 0.58 ng/dL, which had itself been below the reference range, suggesting a substantial individual rise. Taken together, these findings were most consistent with T3-predominant biochemical thyrotoxicosis in the context of levothyroxine overreplacement, a pattern in which excess levothyroxine undergoes peripheral conversion to T3, resulting in TSH suppression and elevated free T3 while free T4 may remain within the population reference range.

**Table 1:** Laboratory values at baseline and admission Marked leukocytosis upon admission with severe hypokalemia and TSH suppression with free T4 elevation from baseline. Baseline values represent the patient's average laboratory results derived from approximately six years of prior medical records.

Parameter	Result	Baseline	Reference Range	Interpretation
Potassium	1.9	4.1	3.5–5.1 mmol/L	Severe hypokalemia
Magnesium	1.8	2.0	1.6–2.6 mg/dL	Within normal limits
Phosphate	2.1	2.7	2.5-4.5 mg/dL	Mild hypophosphatemia
White blood cell	17.7	10.2	4.3–10.6 ×10³/µL	Marked leukocytosis
Alanine transaminase	51	100	<50 U/L	Elevated at baseline
Alkaline phosphatase	144	110	45–117 U/L	Elevated
Thyroid-stimulating hormone (TSH)	<0.100	20.2	0.270–4.200 mIU/L	Suppressed
Free triiodothyronine	5.8	2.9	2.3-4.2 pg/mL	Elevated
Free thyroxine (T4)	1.430	0.58	0.920–1.680 ng/dL	Within normal range (relative increase from baseline)

Given the severity of the patient's neurological deficits on presentation, computed tomography of the brain was obtained to exclude acute intracranial pathology, including stroke or structural lesions, and showed no acute intracranial abnormalities. Neurology and neurosurgery consultations were similarly obtained due to the severity of the motor deficits, to rule out a structural or surgical etiology; both specialties determined the weakness to be metabolic in origin, with no indication for surgical intervention. Leukocytosis was attributed to physiological stress or the corticosteroid effect, but there was no evidence of infection. Endocrinology evaluation recommended discontinuation of levothyroxine therapy due to suppressed TSH and possible iatrogenic thyrotoxicosis. Outpatient follow-up was advised for reassessment of thyroid function. 

The patient was treated with intravenous potassium chloride (60 mEq over six hours) followed by oral supplementation, intravenous normal saline, and magnesium sulfate (2 g) intravenously. Carbohydrate intake was restricted during the acute phase to minimize insulin-driven intracellular potassium uptake. By 18 hours post-admission, muscle strength had fully recovered to 5/5 in all extremities and serum potassium normalized to 4.6-4.7 mmol/L. 

The patient was discharged in stable condition with complete recovery, instructed to avoid corticosteroid exposure without prior endocrinology clearance, and scheduled for thyroid function reassessment within four to six weeks. At outpatient follow-up five weeks later, he reported no recurrent episodes of weakness. Repeat thyroid function testing demonstrated full recovery: TSH 3.8 mIU/L, free T3 3.1 pg/mL, and free T4 0.74 ng/dL, confirming resolution of thyrotoxicosis. Levothyroxine was restarted at a reduced dose of 100 mcg daily with repeat testing scheduled at eight weeks. Serum potassium was 4.3 mmol/L without supplementation. Genetic testing for channelopathies associated with HPP (CACNA1S and SCN4A variants) was offered and accepted, with results pending.

## Discussion

**Figure 1:**
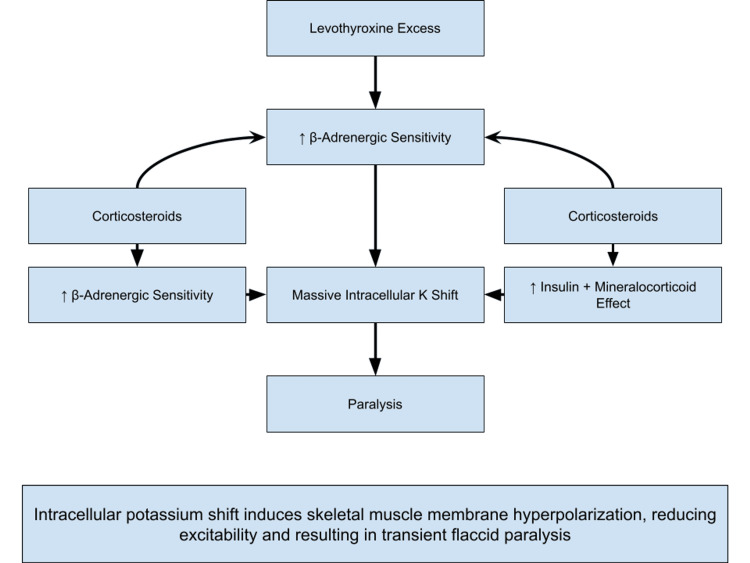
Proposed dual-trigger mechanism of thyrotoxic periodic paralysis (theoretical model based on current pathophysiological understanding). First hit: levothyroxine excess upregulates Na⁺/K⁺-ATPase activity and increases β-adrenergic receptor sensitivity, priming skeletal muscle cells for potassium sequestration. Second hit: corticosteroid administration augments β-adrenergic signaling, induces hyperinsulinemia via hyperglycemia, and exerts mineralocorticoid effects, together driving a massive intracellular potassium shift. The resulting skeletal muscle membrane hyperpolarization manifests as transient flaccid paralysis. This model is proposed based on indirect clinical and mechanistic evidence; direct confirmatory studies in this dual-trigger context are lacking. Figure created by the authors using Canva (Canva Pty Ltd., Sydney, Australia).

This case highlights an unusual presentation of HPP in a patient with treated hypothyroidism who developed iatrogenic thyrotoxicosis from levothyroxine over-replacement, evidenced by a suppressed TSH and an elevated free T3, consistent with T3-predominant thyrotoxicosis. Although the free T4 remained within the population reference range, this pattern is recognized in levothyroxine overreplacement, where excess T4 undergoes preferential peripheral conversion to T3. Clinicians should be aware that a normal free T4 does not exclude functionally significant thyrotoxicosis in this context, and measurement of free T3 is important for complete biochemical characterization. The patient experienced recurrent episodes of proximal-predominant paralysis temporally linked to corticosteroid injections on both occasions, providing strong circumstantial support for the dual-trigger model elaborated below. Hannon et al. (2009) reported a case of TPP caused by excessive L-thyroxine replacement in a Caucasian man with panhypopituitarism. Their case demonstrates that any cause of thyrotoxicosis, including exogenous thyroid hormone excess, can trigger attacks in susceptible individuals [4]. The pathophysiology involves intracellular potassium shifting caused by increased Na⁺/K⁺-ATPase activity, enhanced by both thyroid hormone and corticosteroids (Figure 1) [7]. It must be emphasized, however, that the proposed dual-trigger model represents a mechanistically plausible synthesis of existing evidence rather than a directly confirmed interaction; prospective studies or controlled mechanistic data specifically addressing this combined exposure are currently lacking.

The recurrence of identical episodes following the same corticosteroid agent and dose further supports this interaction and raises the possibility of an underlying genetic susceptibility, such as channelopathies involving CACNA1S or SCN4A variants, which may lower the threshold for paralysis in the setting of thyroid hormone excess [4,7].

This case is noteworthy for several reasons. First, it highlights a rare but important cause of TPP arising from thyroid hormone over-replacement rather than primary thyroid disease, reinforcing that exogenous thyroid hormone excess is a sufficient trigger for TPP in susceptible individuals [4]. Second, the consistent temporal link between intra-articular triamcinolone acetonide 40 mg and each paralytic episode with symptom onset within four hours on both occasions highlights corticosteroids as a significant and often overlooked trigger in vulnerable patients [8]. Triamcinolone's combined glucocorticoid and mineralocorticoid activity, together with its capacity to induce transient hyperglycemia and hyperinsulinemia, provides a plausible mechanistic basis for the precipitated potassium shift [6]. Third, the proximal predominant weakness with greater functional impairment in the upper extremities (2/5 proximally) on formal neurological examination differs from the classic lower limb predominant or ascending pattern typically described in TPP, expanding the recognized clinical spectrum of this condition [9]. While upper extremity involvement is documented in TPP, its predominance as a presenting feature is uncommon and warrants clinical awareness.

From a clinical perspective, this case offers important lessons for patient care. While the proposed mechanism remains to be confirmed prospectively, this case supports the importance of routine monitoring and careful titration of levothyroxine to prevent biochemical thyrotoxicosis, particularly T3-predominant patterns, especially in patients with unexplained episodic weakness. It also underlines the importance of checking for previous episodes of paralysis before administering corticosteroids and advising at-risk patients about possible triggers. In acute cases, proper treatment includes potassium replenishment and nonselective beta-blockers, while long-term management involves maintaining normal thyroid function and avoiding known triggers.

## Conclusions

Overall, this case expands current understanding of HPP by demonstrating that iatrogenic thyroid hormone excess combined with corticosteroid exposure can precipitate recurrent paralytic episodes, even in patients with a history of hypothyroidism. Recognition of this potential dual-trigger mechanism may facilitate earlier diagnosis, prevent recurrence, and guide safer management. In this case, confirmed biochemical resolution of thyrotoxicosis at follow-up and absence of recurrent paralysis after levothyroxine dose reduction further support the causal role of iatrogenic thyroid hormone excess. While more research is needed, these findings suggest that other hypothyroid patients receiving levothyroxine, especially those exposed to corticosteroids, may also be at risk for similar complications. Clinicians should, therefore be aware of this potential interaction to ensure timely intervention and improved outcomes for a broader patient population.
